# The Significance of African Lions for the Financial Viability of Trophy Hunting and the Maintenance of Wild Land

**DOI:** 10.1371/journal.pone.0029332

**Published:** 2012-01-11

**Authors:** Peter Andrew Lindsey, Guy Andrew Balme, Vernon Richard Booth, Neil Midlane

**Affiliations:** 1 Mammal Research Institute, Department of Zoology and Entomology, University of Pretoria, Gauteng, South Africa; 2 Panthera, New York, United States of America; 3 Harare, Zimbabwe; 4 Department of Zoology, University of Cape Town, Cape Town, South Africa; University of Western Ontario, Canada

## Abstract

Recent studies indicate that trophy hunting is impacting negatively on some lion populations, notably in Tanzania. In 2004 there was a proposal to list lions on CITES Appendix I and in 2011 animal-welfare groups petitioned the United States government to list lions as endangered under their Endangered Species Act. Such listings would likely curtail the trophy hunting of lions by limiting the import of lion trophies. Concurrent efforts are underway to encourage the European Union to ban lion trophy imports. We assessed the significance of lions to the financial viability of trophy hunting across five countries to help determine the financial impact and advisability of the proposed trade restrictions. Lion hunts attract the highest mean prices (US$24,000–US$71,000) of all trophy species. Lions generate 5–17% of gross trophy hunting income on national levels, the proportional significance highest in Mozambique, Tanzania, and Zambia. If lion hunting was effectively precluded, trophy hunting could potentially become financially unviable across at least 59,538 km^2^ that could result in a concomitant loss of habitat. However, the loss of lion hunting could have other potentially broader negative impacts including reduction of competitiveness of wildlife-based land uses relative to ecologically unfavourable alternatives. Restrictions on lion hunting may also reduce tolerance for the species among communities where local people benefit from trophy hunting, and may reduce funds available for anti-poaching. If lion off-takes were reduced to recommended maximums (0.5/1000 km^2^), the loss of viability and reduction in profitability would be much lower than if lion hunting was stopped altogether (7,005 km^2^). We recommend that interventions focus on reducing off-takes to sustainable levels, implementing age-based regulations and improving governance of trophy hunting. Such measures could ensure sustainability, while retaining incentives for the conservation of lions and their habitat from hunting.

## Introduction

There is increasing scrutiny on the conservation status of African lions *Panthera leo*. Although few reliable data exist, it is suspected that the continental lion population has declined by at least 30% in recent decades, while the species' geographic range has shrunk by as much as 82% [1]. Key causes for the decline include conflict with pastoralists over livestock, habitat fragmentation, and the loss of available wild prey [2]. Commercial trophy hunting of lions represents an additional potential threat (or opportunity, depending on how it is managed) [3]. Lion populations are particularly sensitive to trophy harvests due to the social disruption and potential for infanticide by incoming males following removal of pride males [4].

Concerns over the impacts of trophy hunting prompted a proposal that lions be listed on CITES Appendix I at the 13^th^ conference of the parties [5]. In theory, such a listing would not necessarily prevent hunting of lions if provision was made under the convention for trophy quotas of the species (as was granted for some leopard *Panthera pardus* and elephant *Loxodonta africana* populations). However, in practice, there is a chance that the US and other importing countries would introduce stricter domestic measures to limit lion trophy imports if the species was listed on CITES Appendix I [6]. There was general opposition to the proposal from the scientific community, due to a belief that declines in lion numbers were not trade-related [5]. There was also recognition among scientists that trophy hunting can create financial incentives for the conservation of lions and their habitats [1]. The CITES proposal was accordingly withdrawn but research has recently emerged suggesting trophy hunting may be more detrimental to lion populations than previously envisaged [7]. Trophy hunting appears to be the primary driver of lion population declines outside (and inside some) protected areas in Tanzania, a country that holds between 30–50% of Africa's lions [3]. Excessive off-takes from trophy hunting also lowered population density of lions, and altered sex-ratios and ranging behaviour of lions in Hwange National Park, Zimbabwe [8], [9], South Luangwa National Park, Zambia [10], and the Bénoué Complex in northern Cameroon [11]. As a result of these findings and due to inherent opposition to sport hunting, a coalition of animal welfare organizations petitioned the US government to list lions as ‘endangered’ pursuant to their Endangered Species Act in 2011 (www.ifaw.org; accessed June 2011). An ESA listing would preclude the importation of lion trophies into the US (the largest market for African trophy hunting; [12], and thus significantly curb trophy hunting of the species, though would not necessarily prevent lions from being killed. In addition, there are concurrent efforts from animal welfare groups to pressure the European Union into banning lion trophy imports (http://www.lionaid.org/campaign/2011/11/recent-press-release-on-our-lion-trophy-import-ban-campaign.htm, accessed November 2011). The recent research findings also suggest that there will be additional pressure in future for an elevated CITES listing for lions.

Prior to implementing far-reaching trade restrictions, an understanding of the potential impacts of such a decision is required. While the direct impacts of trophy hunting on lion populations is increasingly well understood, little is known about the financial significance of lions to trophy hunting, or the potential implications if lion hunting was discontinued. Lions are a key species for trophy hunting due to their iconic status as a member of the ‘big-five’ (a term denoting the five most dangerous African ‘game’ species) and due to the high prices obtained for lion trophies [13]. Consequently, restrictions on the trade of lion trophies may undermine financial incentives for the conservation of lions and their habitats. We assessed the significance of lions to the financial viability of trophy hunting in Africa as a contribution to the debate on the advisability of trade restrictions on lion trophies.

## Methods

### Income earned from trophy hunting

Hunting safaris are traditionally sold as ‘packages’ based on dangerous and charismatic key species (lion, elephant, leopard, buffalo *Syncerus caffer* and rare antelope species) that demand higher prices and longer hunts. Income is accrued through daily rates, which are paid by clients regardless of whether hunts are successful, and trophy fees. We used data from standardized hunt reports (*n* = 267) submitted by clients (www.thehuntingreport.com, accessed June 2011) to establish typical hunt packaging for the five main lion hunting countries (Table 1), and obtained mean prices (daily rates, trophy fees, and the minimum duration of hunts) for packages by surveying operator websites in 2005 (*n* = 114) and 2011 (*n* = 165). Operators were randomly selected from lists of those presenting at US and European hunting conventions and the websites of a minimum of 10–15 from each country sampled (where it was possible to find that number of sites). We compared the prices of key species hunts and assessed changes in prices from 2005–2011. We used the compound US inflation rate to convert 2011 hunt prices into 2005 US dollars, and compared these prices with actual 2005 hunt prices to determine the real increase or decrease in hunt prices during the period.

**Table 1:** Mean price (daily rates, minimum number of days required, trophy fees) and number of key species and plains game typically hunted on safari packages as determined from hunting operator websites and standardized hunt return forms (The Hunting Report website, www.thehuntingreport.com, accessed 2011, June 5).

	Package	Price of hunt packages			Number of animals hunted					
		Daily rate	Min. Days	Trophy fee	Lion	Elephant	Leopard	Buffalo	Sable^b^	PG^a^
Mozambique	Elephant	1,840	21	17,750	0	1.00	0	0.57	0	1.29
*n* = 43	Lion	1,800	18	13,286	1.00	0	0.44	0.44	0.33	3.89
	Leopard	1,821	12	4,444			1.00	0.33	0.17	3.83
	Buffalo	1,408	10	2,734				1.00	0.20	1.90
	Sable	650	10	3,630					1.00	2.33
Namibia	Lion	1,975	20	22,940	1.00	0	0	0	0	5.00
*n* = 31	Elephant	1,617	16	15,875		1.00	0.1	0.20	0	1.20
	Leopard	1,045	14	5,142			1.00	0.20	0	4.00
	Sable	1,427	12	9,125				0.25	1.00	2.50
	Buffalo	1,567	9	6,413				1.00	0	2.13
Tanzania	Lion	3,061	21	11,835	1.00	0.10	0.60	0.70	-	5.20
*n* = 35	Elephant	2,437	20	24,488		1.00	0.40	0.60	-	3.60
	Leopard	2,931	16	8,634			1.00	0.70	-	6.70
	Buffalo	2,198	9	4,331				1.30	-	4.50
Zambia	Lion	2,385	21	5,186	1.00	0	0.10	0.70	0.20	4.90
*n* = 51	Elephant	2,800	14	11,500		1.00	0	1.00	0	7.00
	Leopard	1,709	14	3,550			1.00	0.50	0.50	5.80
	Sable	1,427	12	3,557				1.00	0.30	3.90
	Buffalo	1,389	8	1,781					1.00	5.50
Zimbabwe	Lion	2,050	20	11,714	1.00	0.30	0.60	1.10	0.10	3.60
*n* = 50	Elephant	1,683	18	8,807		1.00	0.20	0.40	0.20	1.50
	Leopard	1,055	15	4,341			1.00	0.60	0.10	4.30
	Buffalo	1096	10	2,774				1.00	0.20	3.80
	Sable	8,90	11	4,409					1.00	5.30

a Plains game;

b Sable were not considered a key species in Tanzania.

Estimates for income earned from trophy hunting in each country were obtained by collecting data on hunting quota and off-takes from as many different hunting areas as possible. In Tanzania, hunting quotas were available from all 143 of the hunting blocks in the country from 2007 (since when, some blocks have been subdivided, taking the total number of blocks in the country to 176, V. Booth unpublished data). Mean percentage utilization of quotas for each species was obtained from the Tanzanian Wildlife Division and applied to quotas in each block to provide an estimate of typical off-takes. In Zimbabwe, data on percentage utilization of quotas were provided by hunting operators for 23 hunting blocks. Mean quota utilization of each species was then applied to the 2011 quota data for all (state owned) safari and forestry areas in Zimbabwe (provided by the Parks and Wildlife Management Authority), and the two largest private conservancies in the country. Community and privately owned hunting blocks (except large conservancies) were excluded from this analysis in Zimbabwe (except for cases where actual off-take data were available) because the status of wildlife in such areas is highly variable so we did not feel confident applying mean quota utilization data to them. For Zambia, 2007 quota data were obtained for the Game Management Areas, excluding a small number of private ranches on which hunting occurs. Mean percentage off-takes (derived from 13 areas, R. Martin unpublished data), were then applied to quotas from Zambian game management areas. In Namibia, data were only available for community conservancies, and so state and privately owned hunting areas were excluded from the analysis (though lions are not hunted in state concessions, and the species only occurs on 8.2% of Namibian farmlands so are rarely hunted, [14]. For Namibian communal conservancies, we only had quota data, and no information was available on the percentage utilization of quotas. Consequently, we used mean quota utilization for each species from the other countries in the analysis. For species that were unique to Namibia, we applied the percentage utilization value from the most ecologically similar species (e.g. for Hartmann's mountain zebra *Equus zebra*, we used the percentage utilization value for plains zebra *Equus burchelli*). In Mozambique, hunting quota data were available for all hunting blocks in the country (from the Ministry of Tourism), but data were excluded for all areas except those for which quota utilization data were available because many hunting areas in that country are severely depleted and quotas bear little resemblance to actual off-takes. Off-take data in Mozambique were limited to the Niassa Reserve (9 blocks, V. Booth unpublished data) and the Coutada 9 and 13 hunting blocks (data provided by operators).

We estimated income (sum of daily rates and trophy fees) accrued per block using off-take data and hunt package prices obtained from the 2011 online survey. We assumed that animals would always be hunted in the most lucrative package available, with excess non-key species hunted in 7-day specialized ‘plains-game’ (primarily antelope) hunts (with the exception of Tanzania and Zambia where plains game hunts are rarely sold). To estimate the total number of packages sold, we multiplied off-take of key species by the mean success rates of hunts (calculated from the hunt return data). This ensured we accounted for daily rates earned from unsuccessful hunts where safaris for key species are paid for by clients, but the target animals are not successfully hunted. To estimate the financial value of each species to a given hunting block, we estimated earnings from trophy fees and daily rates for key species (i.e. those used to sell hunt packages) and non-key species, as follows:

Financial contribution of key species = (% of total trophy fee income comprised by that species*daily rate income from all key species hunts)+(trophy fees from that species*off-take of that species)

Financial contribution of non-key species = (% of total trophy fee income comprised by that species*daily rate income from all plains-game hunts)+(trophy fees from that species*off-take of that species).

### Costs incurred by trophy hunting

Data on the start up and running costs of trophy hunting operations were obtained through a randomized survey of hunting operators at US hunting conventions (Dallas and Houston Safari Clubs, Atlanta Africa Hunting Show), using a structured questionnaire survey following methods outlined in [12] (Table 2). At the shows, an attempt was made to survey every African operator present that sells lion hunts, resulting in coverage of 73.8% of the operators present who offer lion hunts and a sample of *n* = 111 operators. Operators were asked to determine the length of lease of their hunting block(s), and provide an estimate of the total start-up and annual running costs (split into fixed and variable) associated with their hunting operation.

**Table 2:** Costs data used to estimate potential earnings from trophy hunting.

		Start up costs (USD)				
	Typical lease length in years	Minimum total	Mean start up costs/km^2^ ± SE	Maximum total	Running costs (USD)^b^per client day	CorporateTax rate %
Namibia	5	57,000	316±123	440,000	1,067±161	35
Mozambique	27±4^a^	200,000	381±98.8	1,750,000	1,464±328	32
Tanzania	5	150,000	230±108	1,500,000	829±375	35
Zambia^c^	10	50,000	230±108	500,000	829±375	35
Zimbabwe	10	144,000	797±176	1,500,000	1,469±168	25

a In most countries, lease length is largely consistent among blocks, but in Mozambique, due to high variability in this measure, mean lease length ± SE reported by operators was used.

b We assumed (using data from the surveys) that 61±19.1% (mean ± SD) of running costs were fixed, and the remainder were variable.

c Due to a small sample size, we used the Tanzanian mean value for start up and operating costs in Zambia, but used the minimum and maximum values from Zambia.

### Financial Viability

When assessing the viability of hunting operations under different scenarios of lion hunting, we assumed a capital structure of 50% equity and 50% debt for initial investment. Start up costs were calculated by multiplying the concession size by the mean start up costs/km^2^, within bounds set as the minimum and maximum estimates made by operators for start up costs in each country. We split start up costs into lease acquisition costs (60%), camp (10%) and vehicles and equipment (30%). Projected income from trophy hunting was used to calculate income per km^2^ in each hunting area, followed by a mean for each country. We calculated Net Profit Before Tax (NPBT) by subtracting depreciation, interest, and running costs from Revenue. Depreciation on capital investments was calculated by dividing the cost of the investment by the term of the lease, except for vehicles and equipment, which were depreciated over a 5-year period. Interest was calculated at 4.25%, (the US prime interest rate plus one percent, which is the mean rate for commercial loans of medium risk in the US (http://www.federalreserve.gov/Releases/E2/Current/default.htm, accessed July 2011). A standard rate was used for all countries, as hunting operators are often not from the country in which they operate and are thus likely to source funds outside of those countries. Running costs were split into fixed (61%) and variable (39%) based on the mean of estimates from the operator survey, and were converted to a ‘cost per client day’ based on estimated client days in each block (estimated as the number of hunts of each type sold multiplied by the number of days for which such hunt packages are sold). Net Profit After Tax (NPAT) was calculated by reducing NPBT by the country-specific corporate tax rate (assuming NPBT was positive).

For each block, we divided NPAT by the start up costs to calculate Return on Investment (ROI). ROI was compared with a hurdle rate to evaluate financial viability of operations. For this hurdle rate, we used the Weighted Average Cost of Capital (WACC) of a major tourism company, which it uses to evaluate new projects in sub-Saharan Africa, adjusted to accommodate our assumed capital structure and cost of debt, which resulted in a WACC of 6.96%. The adjust WACC of 6.96% is used as the hurdle rate to evaluate financial viability of the hunting operations. We acknowledge that, in addition to financial considerations, there is a “lifestyle” element to the decision to invest in a hunting operation, but we have ignored this for the purposes of our analysis, as it is impossible to quantify. We calculated ROI under three scenarios: i) current lion off-takes, ii) off-takes reduced to 0.5 lions/1000 km^2^ (the recommended sustainable off-take for lions in Tanzania, excluding the Selous Game Reserve; [3], and iii) universal discontinuation of lion hunting. The recommended harvest rates are more conservative than those advised by other authors [15], [16] and would thus likely be safe to apply in other countries.

## Results

**Figure 1:**
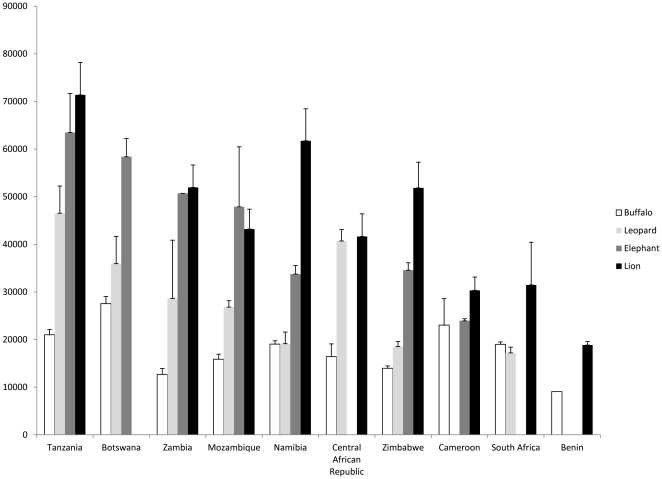
Mean price for the cheapest trophy hunting packages (daily rates and trophy fees) for each of four key species.

The price of hunting packages for key species is influenced by the country and the species involved (F = 13.7, *d.f.* = 2, *p*<0.001, Figure 1); hunts in Tanzania and those involving lions were typically the most expensive (Figure 1). The rate of price increases of key species hunt packages during 2005–2011 varied among species (highest for leopard and lion hunts) and countries (highest in Botswana, and lowest in CAR and Cameroon) (F = 11.8, *d.f* = 2, p<0.001; Table 3).

**Table 3:** Mean annual changes in the price of hunts of key hunting trophies (including trophy fees, and daily rates) during 2005–2011, adjusted for inflation (see footnote).

	Leopard	Lion	Buffalo	Elephant	Mean± SD
Botswana	14.0%	N/A	9.3%	3.7%	9.0±5.1%
Mozambique	5.2%	8.8%	10.5%	1.0%	6.4±4.2%
Namibia	12.8%	4.2%	6.1%	−4.1%	4.7±7.0%
Zambia	2.7%	10.0%	0.4%	No data	4.4±5.1%
Tanzania	1.9%	6.6%	3.7%	4.2%	4.1±1.9%
Zimbabwe	6.0%	7.6%	2.1%	−1.8%	3.5±4.2%
South Africa	4.2%	−1.9%	1.4%	No data	1.2±3.0%
Cameroon	N/A	6.6%	−4.9%	−2.1%	−0.1±6.0%
CAR	N/A	−1.2%	−1.2%	N/A	−1.2±0%
Mean ± SD inc RSA	6.7±4.8%	5.1±4.4%	3.0±4.9%	0.1±3.4%	

We used the compound US inflation rate to convert 2011 hunt prices into 2005 US dollars, and compared these prices with actual 2005 hunt prices to determine the real increase or decrease in hunt prices in the period from 2005 to 2011.

The key species that generate the largest proportion of trophy hunting income are: elephants in Mozambique, Namibia and Zimbabwe, buffaloes in Tanzania, and sable antelopes *Hippotragus niger* in Zambia (Table 4). Mean percentage of overall income that comes from lions is highest in Mozambique, Tanzania, and Zambia (Table 4). If lion off-takes were reduced to 0.5/1000 km^2^, the impact on proportional income from lions would be greatest in Tanzania, Zambia, and Zimbabwe (Table 4). Lions are on quota in the highest proportion of hunting block quotas in Tanzania, Zambia and Mozambique (Table 4).

**Table 4:** Percentage of Income from trophy hunting that is derived from each of a number of key species in several countries.

	Mozambique	Namibia	Tanzania	Zambia	Zimbabwe
Hunting blocks *n*	12	41	138	30	35
% of hunting area in analysis	45.6′	52.9	100	>95	42.4
% of blocks species on quota					
Lion	75.0	26.2	97.8	76.6	52.8
Buffalo	58.3	19.0	99.3	73.3	88.8
Elephant	50.0	47.6	?^b^	10.0	91.7
Leopard	83.3	61.9	100	83.3	91.7
Sable	100	11.9	78.2	53.3	61.1
% of income±SD (rank importance)^a^					
Lion (current off-takes) mean	17.1±13.5 (3)	5.7±13.0 (5)	15.0±5.3 (3)	11.2±7.8 (3)	4.6±5.2 (5)
Lion (0.5/1,000 km^2^) mean	15.0±12.8 (3)	3.6±11.3 (8)	10.6±5.9 (3)	7.5±7.8 (5)	1.37±16.7 (9)
Buffalo mean	13.3±13.4 (5)	4.1±9.4 (7)	**49.0±14.6 (1)**	7.5±9.7 (5)	22.9±11.1 (2)
Elephant mean^b^	**20.6±27.5 (1)**	**27.1±31.8 (1)**	8.5±11.0 (4)	4.2±12.8 (9)	**40.9±18.7 (1)**
Leopard mean	13.7±10.5 (4)	6.1±8.0 (3)	20.2±6.5 (2)	11.2±9.1 (2)	8.5±7.2 (3)
Sable mean	20.0±10.9 (2)	0.3±0.8 (21)	0.4±0.04 (7)	**13.0±18.0 (1)**	5.7±7.8 (4)
Total %comprised of above spp.	84.6±7.6	43.3±0.9	93.1±8.2	46.8±27.8	82.3±14.7

a The rank importance of each species to the earnings from trophy hunting (according to the data from hunting blocks analysed) (including species other than the key species included in the table).

b Data on elephant quotas were unavailable in Tanzania: industry experts advised that approximately 60 elephants are hunted per year and off-takes were assumed to be distributed evenly across blocks excluding those close to the Kenya border where elephants are not hunted.

Estimated gross incomes per km^2^ from trophy hunting are highest in Zimbabwe, Tanzania and Namibia and lowest in Mozambique and Zambia (Table 5). In all countries there was marked disparity between minimum and maximum incomes, related to the size of quotas and off-takes per km^2^ (Table 5). The presence of lions had the greatest proportional impact on incomes in Mozambique and Zambia (Table 5). The presence of lions on quota had a much larger percentage impact on net income (15.0–75.0%) than on gross income (4.2–16.9%, Table 5). Removing lions from quota all together had a much greater impact on gross and net incomes than reductions in lion off-takes to sustainable levels (Table 5).

**Table 5:** Gross and net earnings (US$/km^2^) from trophy hunting with and without lions on quota (± S.E., the ‘with’ scenario includes areas which do not normally have lions on quota – and for those areas, calculations were made without lions on quota for both scenarios).

Earnings/km^2^		Mozambique*n* = 12	Zambia*n* = 30	Tanzania*n* = 138	Namibia*n* = 41	Zimbabwe*n* = 36
	Max	366	2,152	1,838	1,854	128
Gross, with lions	Mean	130±25	148±71	424±31	378±82	1,028±111
	Min	25	3	24	9	2,613
	Max	337	1,977	1,690	1,854	128
Gross, 0.5 lions/1000 km^2^	Mean	125±23	140±65	397±28	358±77	995±108
	Min	25	3	24	9	2,613
	Max	305	1,762	1,663	1,854	128
Gross, without lions	Mean	108±21	126±58	373±28	354±77	985±109
	Min	13	3	19	9	2,613
% reduction with 0.5/1000 km^2^		3.9%	5.4%	6.4%	5.3%	3.2%
% reduction without lions		16.9%	14.9%	12.0%	6.3%	4.2%
	Max	88	1,213	839	983	841
Net, with lions	Mean	−24±23	31.3±43	158±15	120±44	164±40
	Min	−251	−290	−29	−256	−261
	Max	64	1,070	736	983	841
Net, 0.5 lions/1,000 km^2^	Mean	−28±22	25.1±38	139±12	105±44	140±39
	Min	−251	−289	−31	−256	−261
	Max	37	858	717	983	841
Net, without lions	Mean	−42±21	14.7±31	123±13	102±44	133±38
	Min	−263	−290	−34	−256	−261
% reduction with 0.5/1000 km^2^		16.6%	19.8%	12.0%	12.5%	14.6%
% reduction without lions		75.0%	53.0%	22.2%	15.0%	18.9%

Estimated mean returns on investments (ROIs) from trophy hunting were highest in Tanzania, Namibia and Zimbabwe, and were negative in Zambia and Mozambique (Table 6). The majority of hunting blocks in Tanzania and (to a lesser extent) Zimbabwe were estimated to be viable, whereas the majority of those in Zambia and Mozambique were estimated to be unviable regardless of the status of lion hunting. If lion hunting was banned the proportional impact on ROI would be highest in Tanzania and Namibia (Table 6). The impact of closure of lion hunting on the proportion of hunting blocks that are viable would be greatest in Tanzania, Zambia, and Zimbabwe (Table 6). If lion hunting were precluded, trophy hunting could become potentially financially unviable across 43,828 km^2^ in Tanzania, 10,280 km^2^ in Zambia, 3,310 km^2^ in Zimbabwe and 2,120 km^2^ in Mozambique (or 59,538 km^2^ in total – which is equivalent to ∼4 times the area of Serengeti National Park) (Table 6). Reducing off-takes to 0.5 lions/1,000 km^2^, however, would only potentially render trophy hunting financially unviable across 7,005 km^2^ (affecting only Tanzania and Zimbabwe) (Table 6).

**Table 6:** Mean predicted returns on investment from trophy hunting under three lion hunting scenarios, percentages of hunting blocks in which trophy hunting operations are predicted to be financially viable, and the minimum area in which trophy hunting is predicted to be viable (excluding some areas in each country that were excluded from the analyses).

Country (sample size and % of hunting blocks in the country for which analysis was done)/lion hunting scenario	Mean % return on investment ± SD	% of hunting areas viable	Area in which hunting is viable (excluding areas for which data were unavailable) (km^2^)
Mozambique (*n* = 12, 33.2%)			
Current lion off-takes	−7.16±29.7	7.7	2,120
Recommended off-takes	−7.97±20.1	7.7	2,120
Zero off-take	−11.2±19.8	0	0
Namibia (*n* = 41, 34.7%) a			
Current lion off-takes	29.4±90.1	33.3	13,142
Recommended off-takes	24.4±80.0	33.3	13,142
Zero off-take	22.3±79.3	33.3	13,142
Tanzania (*n* = 138, 78.5%)			
Current lion off-takes	37.4±34.9	81.2	146,165
Recommended off-takes	33.1±30.8	79.7	141,960
Zero off-take	28.8±30.8	68.1	102,337
Zambia (*n* = 30, 85.1%) b			
Current lion off-takes	−2.79±32.4	33.3	33,429
Recommended off-takes	−3.69±30.5	33.3	33,429
Zero off-take	−5.82±27.3	23.3	23,149
Zimbabwe (*n* = 36, 43.7%) c			
Current lion off-takes	14.4±21.5	55.6	14,612
Recommended off-takes	12.2±20.5	50.0	11,812
Zero off-take	11.3±20.6	47.2	11,302

a Most of the remainder of Namibia's hunting areas are privately owned and do not support lion populations.

b Including all game management areas (some of which may not actually support hunting in practise), excluding the unknown (but relatively small) area of game ranches in which hunting is practised.

c The remainder of Zimbabwe's hunting areas comprise CAMPFIRE areas (of which lions are hunted in approximately 6,800 km^2^), and private ranches (most of which do not support lions, except for conservancies, which are included in the above-analysis).

## Discussion

### Limitations of our analyses

Key weaknesses in our analyses were estimates of start-up and running costs of hunting operations, where we applied mean values across hunting blocks within each country. In reality, costs vary among blocks due to the varying prices and remoteness of concessions. This weakness affected the accuracy of predictions of profitability of individual blocks, and the proportion of blocks that are estimated to be profitable. However, variation in these parameters did not affect the key conclusion: that the presence/absence of lions on quota affects the proportion of hunting blocks across which trophy hunting is viable.

We used mean percentage utilization of quotas to estimate off-takes, which may have introduced error in estimates of revenue in some blocks. For Namibia, we lacked data on actual off-takes and had to rely on estimates of the proportions of quotas utilized from other countries. For Tanzania and Zambia, the most recent quota data was from 2007 (though the pricing data for all countries was from 2011), and quotas may have changed since then (for example, lion quotas in Zambia were cut in 2011, although the effect on estimated profits is presumably small as percentage utilization would likely increase when quotas are lowered).

The subset of hunting blocks for which we had data may not be entirely representative in some cases: for Namibia and Zimbabwe, private land was largely excluded from the analyses, and in Mozambique we had a relatively small sample of (albeit large) blocks in our analysis. Finally, hunt reports were submitted voluntarily by clients and it is difficult to gauge how representative the sample was of the total number of hunts conducted (for most species, they represented <10% of hunts undertaken annually). However, these were the only standard data available (reporting of hunt composition, duration and success is notoriously poor among statutory authorities) and we confirmed the accuracy of packaging and relative success of hunts with multiple operators from each of the countries assessed. Despite these shortcomings, the data presented provide novel and robust insights into the financial significance of lions to the viability of trophy hunting.

### Pricing of lion hunts

With the exception of rhinoceroses (*Ceratotherium simum* and *Diceros bicornis*) in Namibia and South Africa and exceptionally large elephant trophies, lions generate the highest revenue per hunt of any species in Africa. Prices for lion hunts are particularly high in Tanzania and were also costly in Botswana prior to the moratorium (up to $140,000/hunt, G. Rann, Rann Safaris, pers comm.), presumably because of the renowned trophy quality of Kalahari lions (www.scirecordbook.org; accessed June 2011). The price of lion hunts has increased faster than most species, and this will likely continue as the supply of wild lion trophies declines (3). Lions have the potential to suffer from an anthropogenic Allee effect, where consumers place disproportionate value on rare animals, driving a cycle that could theoretically lead to a species' extinction [17]. However, contrary to that suggestion, the cheapest lion hunts offered are from West Africa where the species is considered Regionally Endangered [18]. The future for major increases in the price of lion hunts is also likely to be undermined by the rapid increase in availability of cheap, high quality trophies from captive-bred lions in South Africa (Lindsey et al. unpublished data) which will assumedly undermine the financial value of wild lions.

### Importance of lions for the financial viability of trophy hunting

#### Mozambique

The proportional financial significance of lion hunting is highest in Mozambique because quotas are low for most other species, lions are on quota in most hunting areas, and few elephants are hunted (32.8±3.0 elephant trophies were exported during 2005–2009, c.f. Botswana 177±22, Zimbabwe 150±43, Tanzania 58.8±14.3, Namibia 30±4.0 and Zambia 3.6±1.8; www.cites.org, accessed April 2011). Most Mozambican hunting areas analyzed appear to generate negative ROI; the presence of lions on quota simply affects the scale of losses. Many Mozambican wildlife areas were depleted during and after the civil war through illegal bush-meat hunting [19], and some hunting operators are investing in unprofitable concessions on the assumption that wildlife populations will recover [20]. While our analysis probably excluded some areas that are profitable in Mozambique (e.g. some game ranches and blocks around the Zambezi Delta; N. Duckworth, Mokore Safaris pers. comm.), the general picture is one of low or negative returns due to depressed wildlife populations. The presence of lions on quota in Mozambique may be important for operators to minimize losses during the rehabilitation of hunting blocks, and to incentivize continued investments. Another key factor limiting the profitability of hunting in Mozambique is that the US Fish and Wildlife Service prohibits the import of Mozambican elephant trophies, and that the CITES export quota for leopards is small (120, c.f. Namibia 250, Tanzania 500, Zambia 300, Zimbabwe 500, www.cites.org, accessed April 2011).

#### Tanzania

Lions generate a large proportion of income from hunting in Tanzania because they are on quota in nearly all hunting blocks, quotas of the species are high, and relatively few elephants are hunted there. If lion hunting were ever banned, there could be severe consequences for the viability of trophy hunting across large areas (∼44,000 km^2^) of Tanzania, which could have serious consequences for wildlife conservation if alternative land uses arose as a result. That said, current profits from trophy hunting in some parts of Tanzania are probably unsustainable due to excessive harvests of lions [3]. Tanzania has recently introduced a 6-year age minimum for lion trophies [21] which would make harvests more sustainable despite uncertainties on the sizes of hunted populations [4]. Nonetheless, it remains to be seen if such a management-intensive system can be effectively applied in a country with a poor record of hunting governance [22]. Sustainability could also be achieved by reducing quotas countrywide to 0.5 lions/1000 km^2^, which was identified as a simple, conservative metric that could be applied to all lion populations to enhance the prospects of achieving sustainability of off-takes [3]. Such a quota reduction would affect the viability of hunting across an area of just ∼4,000 km^2^ and thus would be preferable to a moratorium from a conservation perspective. Alternatively, a short-term moratorium on lion hunting could be considered to allow lion populations to recover, as was implemented in Zimbabwe, followed by reinstatement of trophy hunting based on reduced quotas.

#### Zambia

Most Zambian concessions appear to be running at a loss, probably as a result of the depletion of prey populations due to human settlement and the bush-meat trade in GMAs [23], [24]. In some cases, our methods may have made viable blocks appear unviable by overestimating the start-up costs (we assumed that hunting operators use entire concessions, but in reality many Zambian operators only actually hunt in the portions of GMAs where wildlife persists (C. Burton, S&S Safaris, pers. comm.). Nonetheless, the stark difference in mean returns per unit area between Zambia and neighbouring Zimbabwe provide insight into the effects of inappropriate policies which marginalise communities (which occupy most GMAs) and prevent them from benefitting sufficiently from trophy hunting (thus encouraging illegal harvest for bush-meat) [24] (B. Child, pers comm.).

Lions are relatively significant components of financial returns from trophy hunting in Zambia, due to the low quotas of most species, and low off-takes of elephants. Zambia has a low CITES elephant quota, and their sale is hindered by the fact that the US currently prohibits the import of Zambian elephant trophies. As with Tanzania, a lion hunting ban would potentially undermine the viability of trophy hunting across a large area (10,280 km^2^). Conversely, a reduction of quotas to sustainable levels is not predicted to render trophy hunting unviable in any blocks.

#### Namibia

Lions are of relatively minor importance to the overall financial viability of trophy hunting in Namibia due to the fact that quotas for the species are low. Lion populations, and those of other wildlife, have experienced a marked recovery on Namibian communal land where they coexist with people and their domestic stock [25]. Increased diversity of hunting quotas on Namibian communal conservancies has resulted in increased revenues from hunting, and increased incentives for conservation [26]. Restrictions on hunting of the species may reduce the perceived financial value of lions, encouraging increased retaliatory killings for livestock depredation [27].

#### Zimbabwe

Lions are relatively unimportant for the viability of trophy hunting in Zimbabwe due to the abundance of buffaloes, elephants and leopards on quota, and high quotas of other species. Aside from some conservancies, lions are rarely hunted on private land and so the overall significance of lions to the hunting industry is likely lower than our estimate. Lion off-takes in Zimbabwe are typically well above estimated sustainable levels (Balme et al. unpublished data), with the effect that trophy quality has declined in some blocks and negative population impacts have been observed in Hwange National Park [28]. In response to these trends, a moratorium was imposed on lion hunting in North West Zimbabwe for four years (2005–2008), which combined with the subsequent implementation of sustainable quotas resulted in rapid recovery of lion populations. These experiences highlight the resilience of lion populations and indicate that problems caused by excessive harvests can be rectified if addressed soon enough [28]. In Zimbabwe, a lion hunting moratorium would affect the viability of hunting across ∼3,310 km^2^, whereas quota reductions to sustainable levels would affect viability over an area of 2,800 km^2^.

### Potential for compensating for income from lions

If lion hunting was restricted there would be scope in some places for compensating for lost income through more strategic packaging of quotas, and by increasing quotas and off-takes of other key species. In Tanzania and Zambia, the government imposes tight restrictions on the way in which trophy hunts can be packaged and sold, through dictation of hunt lengths and species compositions of certain packages. If operators were free to market hunts as they wished and in response to market forces, quotas could be sold more efficiently and profitably [29]. Elephant off-takes in Mozambique (32.8/year from a population of 14,079, Blanc et al. 2007), Tanzania (58.8 from 108,816), and Zambia (3.6 from 16,562) are less than the 0.5% that is considered to be sustainable for trophy off-takes and could potentially be increased in some areas [30], [31], [32]. In other cases, trade restrictions on key species imposed by hunting import countries such as the US could be removed to elevate profitability of trophy hunting. However, there is a limit to the extent to which other species can be used to compensate for income from lion hunting as homogenization of the trophy product among countries may compromise the viability in less popular and accessible countries [12].

### Potential conservation implications of reductions in lion hunting

The trophy hunting industry is not dependent on lions for viability in most areas, and other species (notably elephant, buffalo and leopard) are more important in financial terms. However, in a significant minority of hunting areas lions are of key importance, and if hunting of the species was discontinued, hunting operations comprising approximately 59,538 km^2^ could potentially become unviable in the countries assessed, posing a risk that those areas could be lost as lion habitat. This represents 11.5% of the 516,738 km^2^ where lions are currently hunted in the countries included in the analysis (Balme et al. unpublished data), and at least 3.6% of total lion range (1,674,664 km^2^; [33]). Furthermore, lions are hunted across ∼94,000 km^2^ in Central African Republic, ∼7,000 km^2^ in Burkina Faso and ∼4,000 km^2^ in Benin (Balme et al. unpublished data) and inclusion of those countries in the analysis would have likely significantly increased the size of the area across which viability of trophy hunting would be lost if lion hunting was banned (particularly given the low numbers and diversity of other key species hunted in those countries). Even where viability is predicted to be retained, restrictions on lion hunting would affect the overall profitability of trophy hunting and thus reduce the competitiveness of wildlife-based land uses relative to alternatives such as livestock production. Net returns from livestock in semi-arid African rangelands ($10–$30/km^2^/year in areas with 400–800 ml of annual rainfall [34]) are similar to those from trophy hunting in some areas (mean $–24 to $164/km^2^); hence, maximizing returns from hunting is key to ensuring competitiveness of wildlife-based land uses.

In addition to the potential loss of habitat, restrictions on lion hunting could potentially reduce the tolerance of communities in some areas, such as on private land or in Namibian conservancies where land holders are the effective owners of the wildlife resource [25]. Restrictions on lion hunting may also reduce the funds available for management activities such as anti-poaching and community outreach. State budgets for most African parks are below that required to protect them effectively and there is typically little state funding for hunting blocks [35]. In some cases, investments from hunting operators in anti-poaching activities are notable. For example, trophy hunting generates $380–400,000/annum for Niassa National Reserve, almost 20% of the total funds required to maintain the 42,000 km^2^ protected area [36]. Niassa is the focus of lion distribution in Mozambique and the large population (730–1,000 individuals) is believed to be stable or even increasing [37]. Hunting operators in Savé Valley Conservancy (SVC) in Zimbabwe (who removed livestock and reintroduced wildlife, including lions) invest $546,000/annum on anti-poaching and employ 186 permanent scouts, enabling the lion population to increase [38] (Ox Hacking, SVC CEO pers. comm.). Similarly, operators in Coutada 9, Mozambique invest $60,000/annum on anti-poaching, have removed 5,000 gin traps, and have reintroduced lions [20]. However, returns from trophy hunting in most concessions are low, reducing available funds for anti-poaching, regardless of whether lions are hunted. Recent estimates suggest that as much as $1,000/km^2^ may be required to maintain lion populations at a density of at least 50% of their potential carrying capacity (C. Packer, unpublished data) suggesting that hunting may generate a fraction of the funding needed to protect lions effectively in the long term. Similarly, in some countries (notably Tanzania and Zambia), leases of hunting concessions are short, undermining incentives for operators to invest in protecting wildlife. In countries where earnings from hunting are centralized (notably Tanzania and Zambia), wildlife is likely to disappear from hunting blocks in the absence of reform to make communities the primary beneficiaries of trophy hunting (in areas where hunting occurs on community lands) (22, 23, 39).

### Conclusions

While trophy hunting could survive without lion hunting in most areas, the species is an important financial component of an industry which is marginal in some areas and vulnerable to reductions in profitability. Blanket trade restrictions would unfairly punish countries where lion hunting is well managed, and could be negative for lions by undermining the competitiveness of wildlife-based land uses and by undermining tolerance for lions which are typically a high-cost species due to their tendency to kill livestock. A preferable alternative would be the introduction of recommended quotas (0.5 lions/1000 km^2^) as such an intervention would allow lion hunting to be sustainable, while retaining conservation-incentives from trophy hunting. Sustainability would be enhanced further if age-based regulations were implemented (e.g. as in Niassa National Reserve) [37] and if governance of the industry was improved to provide communities with greater stakeholdings. Temporary moratoria on lion hunting could be used to allow recoveries in areas where hunting is implicated in negative lion population trends. Lion populations recover quickly when the pressure of excessive harvests is removed. Consequently, over-hunting is likely to pose little threat to the long term persistence of lions so long as interventions are made to address excessive quotas where they occur. Conversely, if lion hunting was banned, and wildlife-based land uses were replaced by alternatives in some areas, the long term prospects for lion conservation in those areas would be poor and reversing negative trends would be unlikely. Precluding lion hunting may therefore be a greater long term risk to lions than over-hunting. That said, urgent efforts are needed by range states to reform lion hunting management, and temporary moratoria could be considered for use as levers to promote such changes.
